# Reduction of endoplasmic reticulum stress inhibits neointima formation after vascular injury

**DOI:** 10.1038/srep06943

**Published:** 2014-11-06

**Authors:** Shutaro Ishimura, Masato Furuhashi, Tomohiro Mita, Takahiro Fuseya, Yuki Watanabe, Kyoko Hoshina, Nobuaki Kokubu, Katsumi Inoue, Hideaki Yoshida, Tetsuji Miura

**Affiliations:** 1 Department of Cardiovascular, Renal and Metabolic Medicine, Sapporo Medical University School of Medicine, S-1, W-16, Chuo-ku, Sapporo 060-8543, Japan; 2 Department of Laboratory Medicine, Kokura Memorial Hospital, 3-2-1 Asano, Kokurakita-ku, Kitakyushu, Kokura 802-8555, Japan

## Abstract

Endoplasmic reticulum (ER) stress and inappropriate adaptation through the unfolded protein response (UPR) are predominant features of pathological processes. However, little is known about the link between ER stress and endovascular injury. We investigated the involvement of ER stress in neointima hyperplasia after vascular injury. The femoral arteries of 7-8-week-old male mice were subjected to wire-induced vascular injury. After 4 weeks, immunohistological analysis showed that ER stress markers were upregulated in the hyperplastic neointima. Neointima formation was increased by 54.8% in X-box binding protein-1 (XBP1) heterozygous mice, a model of compromised UPR. Knockdown of *Xbp1* in human coronary artery smooth muscle cells (CASMC) *in vitro* promoted cell proliferation and migration. Furthermore, treatment with ER stress reducers, 4-phenylbutyrate (4-PBA) and tauroursodeoxycholic acid (TUDCA), decreased the intima-to-media ratio after wire injury by 50.0% and 72.8%, respectively. Chronic stimulation of CASMC with PDGF-BB activated the UPR, and treatment with 4-PBA and TUDCA significantly suppressed the PDGF-BB-induced ER stress markers in CASMC and the proliferation and migration of CASMC. In conclusion, increased ER stress contributes to neointima formation after vascular injury, while UPR signaling downstream of XBP1 plays a suppressive role. Suppression of ER stress would be a novel strategy against post-angioplasty vascular restenosis.

Restenosis after percutaneous coronary intervention is still a major obstacle in treatment of coronary artery disease. Although drug-eluting stents have proven to be more effective than bare metal stents in reducing the incidence of restenosis after percutaneous coronary intervention (PCI), coronary restenosis and stent thrombosis still remain as significant problems^1^,^2^,^3^,^4^. Therefore, new approaches to these problems in addition to traditional methods for prevention of restenosis, such as administration of immunosuppressants or antiplatelet drugs, are needed. Neointima hyperplasia in coronary lesions subjected to PCI is a pathologic hallmark of restenosis, and it is widely accepted that vascular injury activates vascular smooth muscle cells in the media and induces neointima formation by cell migration into intima and proliferation^5^.

Complex adaptive and maladaptive processes underlie atherosclerosis and response to injury in the coronary artery. Production of proper amounts of functional proteins and their transport are crucial in the adaptive response, and the endoplasmic reticulum (ER) is a vital organelle for quality control of proteins and coordinating the synthesis, folding and trafficking of proteins. Accumulation of unfolded or misfolded proteins in the ER is one of the endogenous sources of cellular stress, known as ER stress. ER stress can occur when cells face physiological processes that demand a high rate of protein synthesis and secretion as well as harmful stresses, including infection, hypoxia, oxidative stress, exposure to a toxic substance and production of mutated proteins. A compensational reaction to ER stress is called an unfolded protein response (UPR), which has mainly three signaling pathways by sensor proteins: PKR-like ER kinase (PERK), activating transcription factor 6 (ATF6) and inositol-requiring enzyme-1α (IRE1α)-X-box binding protein-1 (XBP1) pathways, through the mechanism of arresting translation to alleviate additional sources of ER stress, induction of chaperones to increase protein folding capacity and degradation of unfolded or misfolded proteins^6^.

ER stress and inappropriate adaptation through the UPR are predominant features of pathological processes in several types of tissues. Recent studies have revealed relationships between ER stress and a wide range of diseases, including neurodegenerative disease^7^, cancer^8^, pulmonary artery hypertension^9^ and metabolic disorders^10^. However, little is known about the link between ER stress and endovascular injury. We therefore investigated the involvement of ER stress in neointima hyperplasia after endovascular injury by use of a model of wire-induced femoral artery injury, which mimics vascular remodeling following coronary angioplasty^11^.

## Results

### Involvement of ER stress in neointima formation after vascular injury

**Figure 1:**
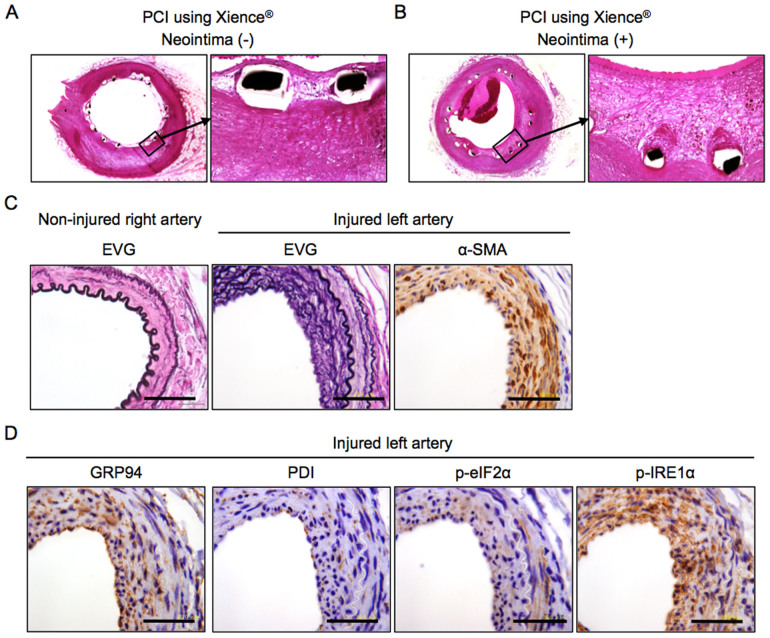
Histological analysis of neointima formation. (A), (B). H-E staining of the coronary artery in an autopsy case (female; 58 years old) of a diabetic patient with angina pectoris who previously had percutaneous coronary intervention (PCI) using a drug-eluting stent, Xience® (Abbott Vascular Japan, Tokyo). Representative images of the coronary artery in the absence (A) and presence (B) of neointima formation after stenting are shown. Strut thickness: 81 µm. (C). Left femoral arteries of 7-8-week-old male mice were subjected to wire-induced vascular injury. After 4 weeks, Elastica-Van Gieson (EVG) staining was performed in both the non-injured right and injured left femoral arteries, and histological staining was performed using α-SMA antibody in the wire-injured left femoral artery. (D). Histological staining was performed using antibodies of GRP94, PDI, phospho-eIF2α (p-eIF2α) and phospho-IRE1α (p-IRE1α) in the wire-injured left femoral artery. Scale bars: 50 µm.

Representative staining of the coronary artery after PCI using a drug-eluting stent (Xience®; Abbott Vascular, Tokyo, Japan) in an autopsy case (female; 58 years old) is shown in Figure 1A and 1B. Neointima formation after angioplasty was prevented by a drug-eluting stent in a coronary lesion (Figure 1A) but not in another stenotic lesion in the same patient (Figure 1B), indicating that restenosis after angioplasty is not totally eradicated by drug-eluting stents.

Neointima formation in the coronary artery after PCI could be mimicked in the femoral arteries of mice. The left femoral arteries of 7–8-week-old male mice were subjected to wire-induced vascular injury. Four weeks later, neointima formation mainly consisting of vascular smooth muscle cells, which was confirmed by staining of α-smooth muscle actin (α-SMA), had developed in the wire-injured left femoral artery (Figure 1C). In the non-injured right femoral artery, no neointima formation was observed. Immunohistological analysis for ER stress markers showed that glucose-regulated protein 94 (GRP94), protein disulfide isomerase (PDI) and phosphorylation of eukaryotic initiation factor 2 α (eIF2α) (p-eIF2α) and IRE1α (p-IRE1α) were present in the hyperplastic neointima in injured regions (Figure 1D).

### Effect of *Xbp1-*haploinsufficiency on neointima formation

**Figure 2:**
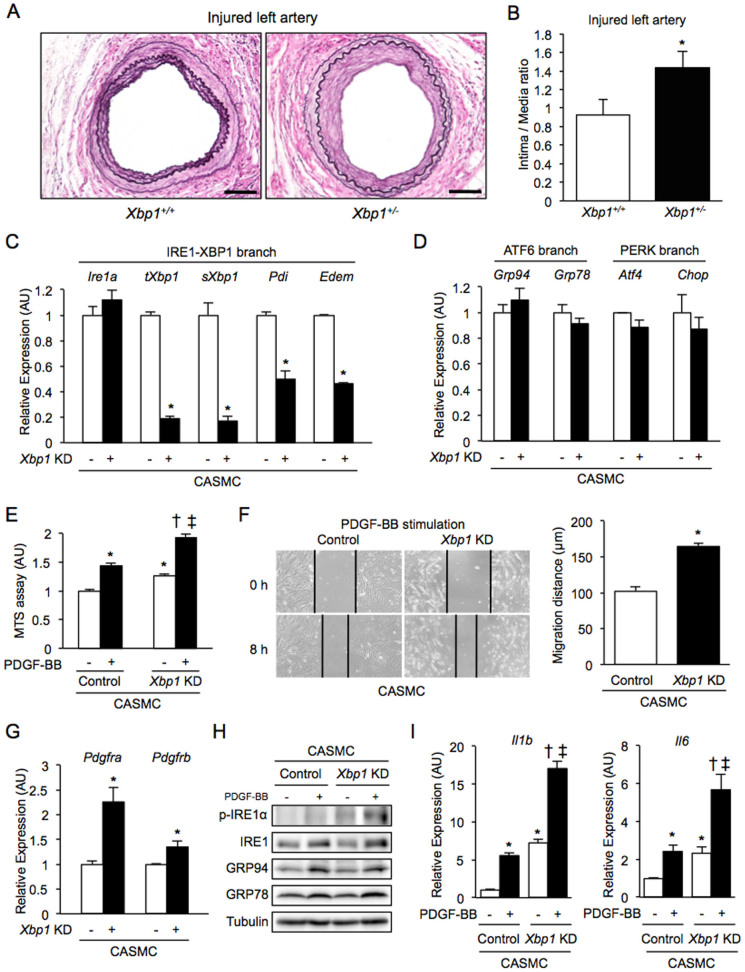
Neointima formation, cell proliferation, cell migration and inflammation in *Xbp1* haploinsufficiency. (A), (B). Left femoral arteries of 7-8-week-old male *Xbp1^+/+^* (n = 7) and *Xbp1^+/−^* (n = 7) mice were subjected to wire-induced vascular injury. After 4 weeks, EVG staining was performed in the left femoral artery (A). Scale bars: 50 µm. The extent of neointima formation was evaluated as intima-to-media ratio in the wire-injured artery of *Xbp1^+/+^* and *Xbp1^+/−^* mice (B). *P < 0.05. (C), (D). Gene expression of unfolded protein response (UPR) markers in the IRE1α-XBP1 branch (C) and the other branches (D) in *Xbp1*-knockdown human coronary artery smooth muscle cells (CASMC). *P < 0.05. (E). Cell proliferation was assessed by an MTS assay in *Xbp1*-knockdown CASMC with 20 ng/ml platelet-derived growth factor-BB (PDGF-BB) for 48 h. *P < 0.05 vs. Control-PDGF-BB(-); †P < 0.05 vs. *Xbp1* KD-PDGF-BB(-); ‡P < 0.05 vs. Control-PDGF-BB(+). (F). Cell migration was assessed by a scratch wound assay in *Xbp1*-knockdown CASMC treated with 20 ng/ml PDGF-BB for 8 h. Photographs were taken, and migration distance was measured by ImageJ. *P < 0.05. (G). Gene expression of PDGF receptors, PDGFR-α and PDGFR-β, in *Xbp1*-knockdown CASMC. *P < 0.05. (H). Western blotting analysis of ER stress markers in *Xbp1*-knockdown CASMC treated with 20 ng/ml PDGF-BB for 48 h. (I). Gene expression of inflammatory markers, IL-1β and IL-6, in *Xbp1*-knockdown CASMC treated with 20 ng/ml PDGF-BB for 48 h. *P < 0.05 vs. Control-PDGF-BB(−); †P < 0.05 vs. *Xbp1* KD-PDGF-BB(−); ‡P < 0.05 vs. Control-PDGF-BB(+).

To further investigate the association between vascular remodeling and ER stress *in vivo*, wire injury-mediated neointima hyperplasia was induced in XBP1 heterozygous (*Xbp1^+/−^*) mice as a model of compromised UPR. Thickness of the neointima after wire injury was significantly larger (by 54.8%) in the *Xbp1^+/−^* mice than in wild-type mice (Figure 2A, B).

The mechanism by which XBP1 deletion promoted neointima formation was investigated by using human coronary artery smooth muscle cells (CASMC). In CASMC, siRNA-mediated *Xbp1*-knockdown resulted in efficient gene suppression of total XBP1 (*tXbp1*) and the spliced form of XBP1 (*sXbp1*) by 81.1% and 83.0%, respectively (Figure 2C). Knockdown of *Xbp1* in CASMC significantly decreased gene expression of PDI and ER degradation-enhancing α-mannosidase-like protein (EDEM), downstream molecules of XBP1, whereas gene expression of IRE1α, an upstream molecule of XBP1, was not significantly changed (Figure 2C). On the other hand, *Xbp1*-knockdown in CASMC did not significantly change gene expression of GRP94 and GRP78, downstream of the ATF6 branch, or activating transcription factor 4 (ATF4) and CCAAT/enhancer binding protein homologous protein (CHOP), downstream of the PERK branch (Figure 2D).

An MTS assay showed that *Xbp1-*knockdown in CASMC significantly promoted basal and platelet-derived growth factor (PDGF)-BB-induced cell proliferation compared with the controls (Figure 2E). A scratch wound assay showed that PDGF-BB-induced cell migration was significantly increased in *Xbp1-*knockdown CASMC compared with that in control cells (Figure 2F). Gene expression levels of PDGF receptor-α (PDGFR-α) and PDGF receptor-β (PDGFR-β) were significantly higher in *Xbp1*-knockdown CASMC than in the controls (Figure 2G). In addition, knockdown of *Xbp1* significantly increased phosphorylation of IRE1α upstream of XBP1 but not GRP94 or GRP78 downstream of the ATF6 branch upon chronic stimulation with PDGF-BB at the dose of 20 ng/ml for 48 h (Figure 2H). Furthermore, knockdown of *Xbp1* significantly increased gene expression levels of inflammatory cytokines, including interleukin-1β (IL-1β) and interleukin-6 (IL-6), in a basal condition and further increased those gene expression levels upon chronic stimulation with PDGF-BB in CASMC (Figure 2I).

### Effect of ER stress reducers on neointima formation

**Figure 3:**
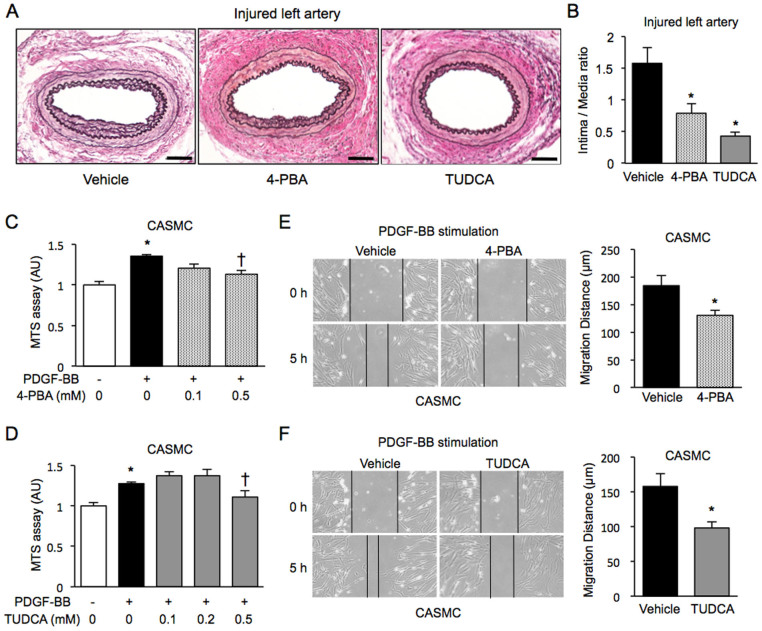
Effects of chemical chaperones on neointima formation, cell proliferation and cell migration. (A), (B). Representative staining of EVG in the wired-injured left femoral arteries of mice treated with daily intraperitoneal injection of a vehicle (n = 11), 100 mg/kg 4-PBA (n = 8) or 500 mg/kg TUDCA (n = 6) for 4 weeks (A). Scale bars: 50 µm. The extent of neointima formation was evaluated as intima-to-media ratio in the wire-injured femoral artery (B). *P < 0.05 vs. Vehicle. (C), (D). Cell proliferation was assessed by an MTS assay in chronic stimulation with 20 ng/ml PDGF-BB for 48 h in CASMC treated with 0-0.5 mM of 4-PBA (C) or 0–0.5 mM of TUDCA (D). *P < 0.05 vs. PDGF-BB(−); †P < 0.05 vs. PDGF-BB(+). (E), (F). Cell migration was assessed by a scratch wound assay in CASMC treated with 0.5 mM 4-PBA (E) or 0.5 mM TUDCA (F) upon stimulation with PDGF-BB for 5 h. Photographs were taken, and migration distance was measured by ImageJ. *P < 0.05.

Treatment with ER stress reducers known as chemical chaperones, 4-phenylbutyric acid (4-PBA) and tauroursodeoxycholic acid (TUDCA), for 4 weeks did not affect media thickness but significantly decreased intima thickness, resulting in a reduction in the intima-to-media ratio, a severity index of neointima formation, by 50.0% and 72.8%, respectively, compared with those after vehicle treatment (Figure 3A, B).

Significant proliferation of CASMC by treatment with 20–80 ng/ml PDGF-BB for 48 h (Figure S1) and absence of toxicity of treatment with 4-PBA (Figure S2A) or TUDCA (Figure S2B) at the dose of up to 0.5 mM for 48 h in CASMC were confirmed in an MTS assay. Proliferation of CASMC was induced by chronic stimulation with 20 ng/ml PDGF-BB for 48 h, and treatment with 0.5 mM 4-PBA (Figure 3C) or 0.5 mM TUDCA (Figure 3D) significantly inhibited PDGF-BB-induced cell proliferation. A scratch wound assay showed that both 0.5 mM 4-PBA and 0.5 mM TUDCA significantly suppressed cell migration after stimulation with 20 ng/ml PDGF-BB for 5 h (Figure 3E, F) as well as serum stimulation for 5 h (Figure S3A, B).

### Effect of ER stress reducers on UPR and inflammation in CASMC

**Figure 4:**
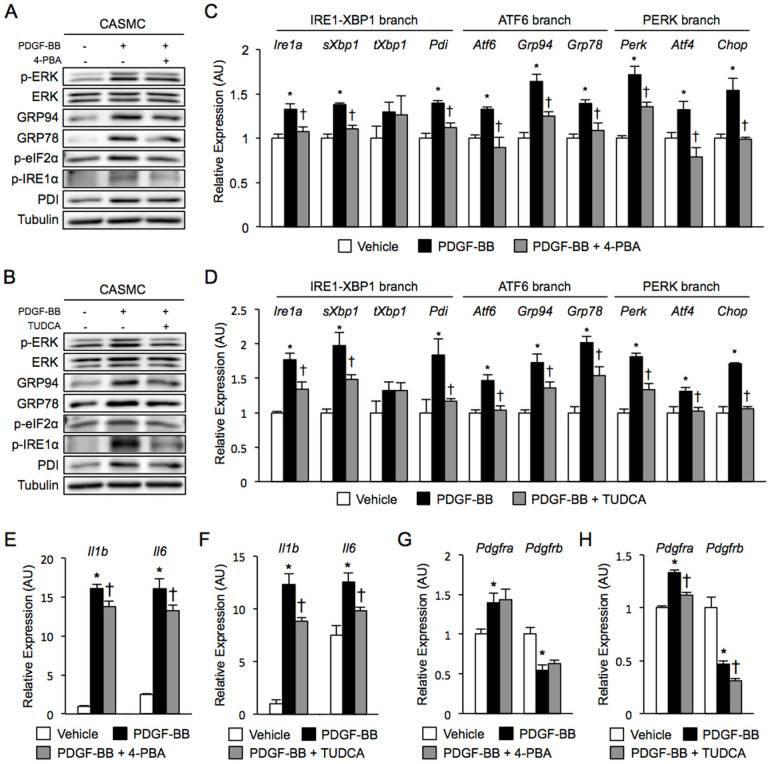
Effects of chemical chaperones on PDGF-BB-induced UPR activation and inflammation in CASMC. (A), (B). Western blotting analysis of ER stress markers in CASMC treated with 0.5 mM 4-PBA (A) and 0.5 mM TUDCA (B) upon chronic stimulation with 20 ng/ml PDGF-BB for 48 h. (C–H). Gene expression analysis of ER stress markers (C, D), inflammatory cytokines (E, F) and PDGF receptors (G, H) in CASMC treated with 0.5 mM 4-PBA and 0.5 mM TUDCA, respectively, upon chronic stimulation with 20 ng/ml PDGF-BB for 48 h. *P < 0.05 vs. Vehicle, †P < 0.05 vs. PDGF-BB.

Chronic stimulation of CASMC with 20 ng/ml PDGF-BB for 48 h led to phosphorylation of extracellular signal-regulated kinase (ERK) and activation of the UPR as indicated by phosphorylation of IRE1α and eIF2α and induction of GRP94 and GRP78 (Figure 4A, B) as well as elevation of mRNA levels of *Ire1a*, *sXbp1* and *Pdi* in the IRE1α-XBP1 branch, *Grp94* and *Grp78* in the ATF6 branch, and *Perk*, *Atf4* and *Chop* in the PERK branch (Figure 4C, D).

Similarly, but weakly, chronic stimulation of CASMC with 20 ng/ml PDGF-AA for 48 h induced gene expression of ER stress markers and activation of ERK and the UPR (Figure S4A, B). Treatment with 0.5 mM 4-PBA or 0.5 mM TUDCA significantly suppressed PDGF-BB-induced elevation of protein and mRNA levels of ER stress markers in CASMC (Figure 4A–D). Moreover, chronic stimulation of CASMC with PDGF-BB (Figure 4E, F) or PDGF-AA (Figure S4C) significantly increased expression levels of inflammatory genes, including *Il1b* and *Il6*, and the PDGF-BB-induced inflammatory genes were reduced by treatment with 4-PBA (Figure 4E) or TUDCA (Figure 4F). Chronic treatment of CASMC with PDGF-BB (Figure 4G, H) or PDGF-AA (Figure S4D) for 48 h significantly increased gene expression of *Pdgfra* and conversely down-regulated gene expression of *Pdgfrb*. Treatment with TUDCA, but not PBA, decreased gene expression levels of PDGF receptors (Figure 4G, H).

## Discussion

We demonstrated for the first time that activation of all branches of the UPR, including IRE1α-XBP1, ATF6 and PERK pathways, and accompanying inflammation are associated with neointima formation in the artery after wire injury. Neointima thickening was augmented in *Xbp1^+/−^* mice, and proliferative and migratory responses of CASMC to PDGF-BB *in vitro* were enhanced by knockdown of *Xbp1*. The augmented proliferation of CASMC by knockdown of *Xbp1* was associated with enhanced signaling (phosphorylation) of IRE1α, a molecule upstream of XBP1, as well as induction of inflammation. In contrast, overall suppression of UPRs by use of 4-PBA or TUDCA suppressed neointima hyperplasia after wire injury *in vivo* and proliferation and migration of CASMAC *in vitro*. These findings suggest that ER stress contributes to vascular remodeling following intravascular injury, while UPR signaling downstream of XBP1 plays a suppressive role in neointima formation.

The pathology of neointima formation is traditionally thought to be migration and proliferation in the media, which are affected by various elements derived from around immune and vascular cells, such as growth factors and inflammatory cytokines^5^. Recent studies have also shown that smooth muscle cell-like cells differentiated from bone marrow-derived progenitor cells play important roles in neointima formation^12^,^13^. PDGFs have a prominent role in the migration of smooth muscle cells into the neointima following acute injury and atherosclerosis^14^. PDGF-BB can bind to both the PDGFR-α and PDGFR-β, whereas PDGF-AA can only bind to the PDGFR-α^14^, suggesting there might be different in the significance of neointima formation. UPR activation and increased gene expression of inflammatory genes in CASMC were similar between chronic stimulation of PDGF-BB and PDGF-AA, but the effect seemed to be stronger in the treatment with PDGF-BB than PDGF-AA (Figure 4, S4). Since it has been reported that activation of PDGF-BB is involved in neointima formation after endovascular injury^14^,^15^, we mainly examined roles of ER stress in responses of proliferation and migration functions in CASMC to PDGF-BB in the present study. PDGF-BB-induced proliferation of CASMC and enhanced migratory function were accompanied by upregulation of UPR markers. These effects of PDGF-BB on CASMC were significantly suppressed by 4-PBA or TUDCA, indicating that ER stress was causally related to proliferation/migration of CASMC.

It has been shown that activation of UPR itself induces inflammation through c-Jun N-terminal kinase (JNK)-activator protein-1 (AP-1) and inhibitor of κ B (IκB) kinase (IKK)-nuclear factor-κ B (NF-κB) pathways^16^,^17^,^18^: IRE1α phosphorylates JNK and IKK, whereas the PERK-eIF2α pathway attenuates IκB, resulting in NF-κB activation. Activation of NF-κB has been reported to promote vascular smooth muscle cell proliferation and neointima formation^19^,^20^,^21^,^22^. Therefore, prolonged UPR activation under the condition of proliferation of vascular smooth muscle cells would cause vascular inflammation, leading to the development and acceleration of neointima formation. Moreover, it is well known that chronic inflammation *per se* induces ER stress^23^. Thus, a vicious cycle of prolonged UPR and chronic inflammation might underlie persistent proliferation of smooth muscle cells and neointima formation after wire injury. This notion is supported by the finding that expression levels of inflammatory cytokines, including IL-1β and IL-6, were increased with activation of UPR upon chronic stimulation with PDGF-BB (Figures 2, 4).

Reduction of XBP1 expression enhanced neointima hyperplasia after wire injury *in vivo* (Figure 2A, B) and proliferation/migration of CASMC in response to PDGF-BB *in vitro* (Figure 2E, F). The altered responses of smooth muscle cells by partial deletion of *Xbp1* might be attributable to reduced PDI- and EDEM-mediated signals downstream of XBP1 and/or enhanced IRE1α-JNK- and IRE1α-IKK-mediated activation of inflammation by a feedback signal to upstream of XBP1. In fact, we showed augmentation of IRE1α activation and gene induction of inflammatory cytokines by knockdown of *Xbp1* in CASMC (Figure 2h, I).

Interestingly, expression levels of PDGF receptors, PDGFR-α and PDGFR-β, were increased in *Xbp1*-knockdown CASMC compared with the levels in control cells (Figure 2G). Furthermore, chronic treatment of CASMC with PDGF-BB significantly increased gene expression of PDGFR-α, though gene expression of PDGFR-β was down-regulated by ligands (Figure 4G, 4H, S4D). Increased expression levels of PDGF and its receptors, PDGFR-α and PDGFR-β, in atherosclerotic lesions compared with those in the normal vessel wall have been reported^14^. An important role of PDGFR-β in arterial injury disorders and potential benefit of its modulation for preventing hyperplasia of arterial smooth muscle cells have been shown^24^,^25^. Recent studies have also demonstrated that PDGFR-α promoted proliferation of vascular smooth muscle cells^26^,^27^,^28^. Although there has been no report regarding the association between PDGFRs and UPR, expression of PDGFR-α has been shown to be upregualted by IL-1β via augmentation of CCAAT/enhancer binding protein δ (C/EBPδ) expression^29^,^30^. In the present study, knockdown of *Xbp1* increased the expression of IL-1β as well as PDGF receptors. In previous studies, expression of C/EBPδ was enhanced in IRE1α-null mice and *Ire1a*-knockdown hepatocytes^31^, and ER stress increased the production of IL-1β in pancreatic β cells and THP-1 cells through IRE1α and PERK pathways^32^. Collectively, these findings support the notion that C/EBPδ-mediated increase in PDGFR-α expression by IL-1β is involved in smooth muscle cell hyperplasia by partial deletion of XBP1.

In the present study, we revealed beneficial effects of chemical chaperones, 4-PBA and TUDCA, on both wire injury-induced neointima formation in the artery *in vivo* and on proliferation, migration and inflammatory response of CASMC *in vitro*. Chemical chaperones are thought to reduce ER stress through stabilizing misfolded proteins, reducing their aggregation, facilitating escape of mutant proteins from the cell's control systems and altering the activity of endogenous molecular chaperones. Both 4-PBA and TUDCA have pharmacological actions other than those as chemical chaperones, such as inhibition of histone deacetylase by 4-PBA and stabilization of the mitochondrial membrane by TUDCA^33^. However, our observations that two structurally different chemical chaperones had similar impacts on smooth muscle cells *in vivo* and *in vitro* except for gene expression of PDGF receptors strongly indicate that suppression of ER stress was a mechanism of their actions on neointima formation after vascular injury.

In conclusion, activation of all branches of the UPR, including IRE1α-XBP1, ATF6 and PERK pathways, is associated with neointima formation after vascular injury and with PDGF-BB-induced proliferation, migration and inflammation in vascular smooth muscle cells. The responses of smooth muscle cells to wire injury *in vivo* and to PDGF-BB *in vitro* were augmented by partial deletion of XBP1 but were significantly attenuated by chemical chaperones. The results suggest that the use of a chemical chaperone is beneficial for prevention of post-angioplasty vascular restenosis, though UPR signaling downstream of XBP1 plays some suppressive role in neointima formation.

## Methods

### Coronary artery specimens

A set of coronary artery specimens of an autopsy case was obtained in accordance with the Institutional Review Board of the Kokura Memorial Hospital and Sapporo Medical University. Written informed consent was received from bereaved family members.

### Biochemical reagents and animals

All experimental protocols were approved by the Animal Care Committee of Sapporo Medical University, and animal care and experimental procedures performed in accordance with the Animal Care Committee of Sapporo Medical University. All biochemical reagents were purchased from Sigma-Aldrich (Saint Louis, MO) unless indicated otherwise. Male C57BL/6J mice were obtained from Oriental Yeast (Tokyo, Japan). XBP1 heterozygous (*Xbp1^+/−^*) mice were generated in the laboratory of Dr. Laurie H. Glimcher (Weill Cornell Medical College). They were backcrossed for more than 8 generations into the C56BL/6J genetic background. Mice were kept on a 12-hour light cycle in a pathogen-free barrier facility and were placed on a regular chow, *ad libitum*.

### Wire injury model

Male mice aged 7–8 weeks were anesthetized with an intraperitoneal injection of pentobarbital (50 mg/kg), and endoluminal injury of the left femoral artery was performed as previously described^11^. In brief, the left femoral artery was exposed and clamped upstream and downstream of the artery. From the branch of the femoral artery, a wire (diameter: 0.014 inches) was inserted into the artery and removed from the vessel followed by branch ligation. Treatment with intraperitoneal injection of 100 mg/kg 4-PBA (Alpha Aesar, Heysham, UK), 500 mg/kg TUDCA (Tokyo Chemical Industry, Tokyo, Japan) or a vehicle was performed once a day for 4 weeks.

### Histological analysis

At 4 weeks after wire injury, mice were anesthetized and fixed by perfusion with saline followed by 10% formalin through a cannula placed in the left ventricle. Both femoral arteries were excised from each mouse and embedded in paraffin. Neointima formation in the femoral arteries was evaluated at ten locations at 100-µm intervals, with the most distal site located at the origin of the branch through which the wire had been inserted. The sections were stained by the Elastica-Van Gieson (EVG) protocol. Images of arteries were captured with a digital color camera (DP21; Olympus, Tokyo, Japan) mounted on a microscope (CX41; Olympus). Quantitative analysis for areas of the intima and media was performed using ImageJ software. All of the measurements were performed in a double-blind manner by two different researchers. For immunohistochemical analysis, sections were incubated overnight at 4°C with primary antibodies against α-SMA (Dako, High Wycombe, UK), GRP94 (Enzo Life Science, Farmingdale, NY), PDI (Cell Signaling, Danvers, MA), phospho-eIF2α (Invitrogen, Carlsbad, CA) and phospho-IRE1α (Novus Biologicals, Littleton, CO). The sections were treated with rabbit biotinylated antibodies against rat IgG for 1 h at room temperature followed by incubation with avidin-biotin complex (Vectastain Elite ABC; Vector Laboratories, Burlingame, CA) and visualized with ImmPACT DAB peroxidase substrate. Slides were counterstained with hematoxylin.

### Cell culture

Human coronary artery smooth muscle cells (CASMC) were purchased from Lonza Inc. (Walkersville, MD) and were grown in a smooth muscle growth medium (SmGM; Lonza) including smooth muscle basal medium (SmBM) with 5% fetal bovine serum (FBS) according to the manufacturer's instructions. After serum starvation for 24 h, CASMC were stimulated with SmBM containing 0–80 ng/ml PDGF-BB or 20 ng/ml PDGF-AA (R&D, Minneapolis, MN) in the presence of 0–0.5 mM 4-PBA or 0–0.5 mM TUDCA for 48 h.

### Small interfering RNA knockdown

Small interfering RNA (siRNA) analysis was performed by using Stealth RNAi (Invitrogen) targeting *Xbp1* mRNA (Cat. #10620319, AAG GGC AUU UGA AGA ACA UGA CUG G) and negative control (Cat. #12935-200). CASMC were transfected with specific or control Stealth RNAi by lipofectamine 2000 (Invitrogen) and cultured for 24 h.

### Cell proliferation and migration assays

An MTS assay was performed for assessing cell proliferation using the cell titer Aqueous One Solution Proliferation Assay (Promega, Madison, WI). After serum starvation for 24 h, CASMC were stimulated with SmBM containing 20 ng/ml PDGF-BB in the presence of 0–0.5 mM 4-PBA or 0–0.5 mM TUDCA for 48 h. A scratch wound assay was performed for determining cell migration. After overnight serum starvation, CASMC were scratched with a sterile pipette tip to produce a straight cell-free zone. Cells were stimulated with 20 ng/ml PDGF-BB or 5% serum in the presence and absence of 0.5 mM 4-PBA or 0.5 mM TUDCA. Pictures were taken at baseline and after stimulation with PDGF-BB for 5–8 h or serum for 5 h. Migration distance was measured using ImageJ software.

### Quantitative real-time PCR

Total RNA was isolated using Trizol Reagent (Invitrogen). One μg of total RNA was reverse-transcribed by using the high capacity cDNA archive kit (Applied Biosystems, Foster City, CA). Quantitative real-time PCR analysis was performed using SYBR Green in the real-time PCR system (Applied Biosystems, Warrington, UK). The thermal cycling program was 10 min at 95°C for enzyme activation and 40 cycles of denaturation for 15 s at 95°C, 30-s annealing at 58°C and 30-s extension at 72°C. Primers used in the present study are listed in Table S1. To normalize expression data, 18s rRNA was used as an internal control gene.

### Western blotting

Total protein content of the samples was assessed by a microplate protein assay (Bio-Rad, Hercules, CA), and equal amounts of protein per sample and known molecular weight markers were subjected to SDS–polyacrylamide gel electrophoresis (SDS-PAGE). Proteins were electrophoretically transferred onto PVDF membranes (Whatman, Florham Park, NJ) and incubated for 1 h at room temperature with a blocking solution (3% bovine serum albumin) in Tris-buffered saline buffer containing 0.1% Tween 20 (TBST). The blocked membranes were incubated with primary antibodies for ERK, phospho-ERK, IRE1α and PDI (Cell Signaling), GRP94 (Enzo Life Science), GRP78 (BD Japan), phospho-eIF2α (Ser52) (Invitrogen), phospho-IRE1α (Ser724) (Novus Biological) and β-tubulin (Santa Cruz Biotechnology, Santa Cruz, CA) overnight at 4°C and washed three times with TBST. The membranes were incubated with a secondary antibody conjugated with horseradish peroxidase (GE Healthcare, UK) for 1 h at room temperature and washed. Immunodetection analyses were performed using a BM Chemiluminescence Blotting Substrate (POD) Kit (Roche Diagnostics, Mannheim, Germany).

### Statistical analysis

Experimental results are expressed as means ± SEM. Differences of means were analyzed by using one-way ANOVA with the Turkey-Kramer post-hoc test for multiple groups and unpaired Student's *t*-test for two groups. Probability values < 0.05 were considered significant.

## Author Contributions

S.I. and M.F. designed the project; S.I., M.F., T.Mita, T.F., Y.W., K.H., N.K., K.I. and H.Y. performed experiments; S.I., M.F., T.Mita, T.F., Y.W., K.H., N.K., K.I. H.Y. and T.Miura analyzed and interpreted data; and S.I., M.F. and T.Miura prepared the manuscript.

## Supplementary Material

Supplementary Information Supplementary information
