# Supplementary material for: Reduction of endoplasmic reticulum stress inhibits neointima formation after vascular injury
Source: Sci Rep. 2014 Nov 6;4:6943. doi: 10.1038/srep06943 (PMC4221790; doi:10.1038/srep06943)
Supplement: Supplementary Information [file srep06943-s1.pdf]

Supplementary information

Reduction of endoplasmic reticulum stress inhibits neointima formation after vascular injury

Shutaro Ishimura*, Masato Furuhashi*, Tomohiro Mita, Takahiro Fuseya, Yuki Watanabe, Kyoko Hoshina, Nobuaki Kokubu, Katsumi Inoue, Hideaki Yoshida, Tetsuji Miura
**The first two authors equally contributed to this work.*

Figure S1

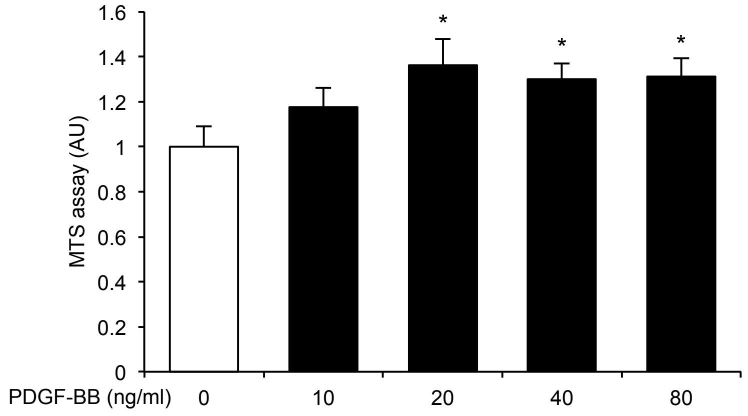

Figure S1. Dose dependent effect of PDGF-BB on cell proliferation in CASMC. Cell proliferation was assessed by an MTS assay in CASMC treated with 0-80 ng/ml PDGF-BB. *P < 0.05.

Figure S2

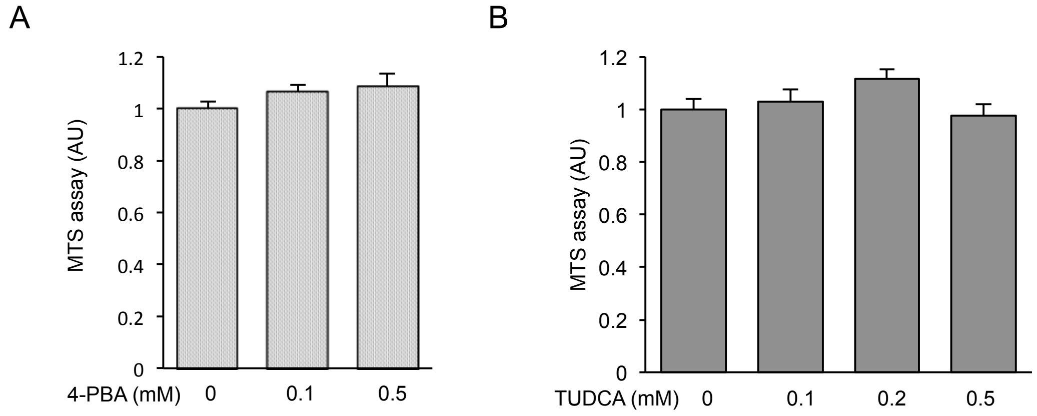

Figure S2. Cell toxicity of 4-PBA and TUDCA in CASMC.
A, B. Cell toxicity was assessed by an MTS assay in CASMC treated with 0-0.5 mM 4-PBA (A) or 0-0.5 mM TUDCA (B).

Figure S3

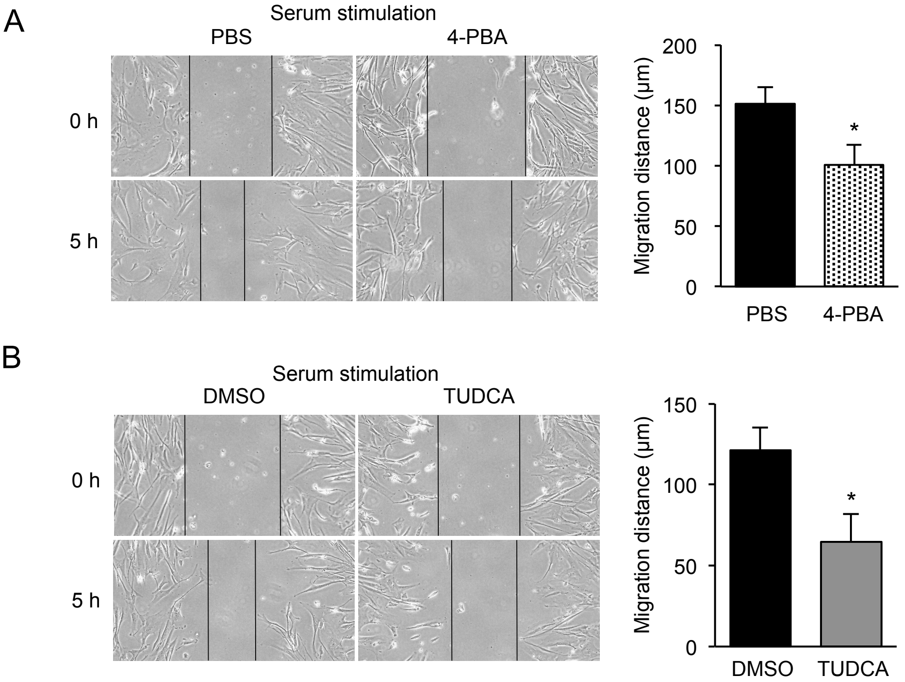

Figure S3. Effect of chemical chaperones on cell migration induced by serum stimulation in CASMC.

A, B. Cell migration was assessed by a scratch wound assay in CASMC treated with 0.5 mM 4-PBA (A) or 0.5 mM TUDCA (B) upon stimulation with 5% serum for 5 h. Photographs were taken, and migration distance was measured by ImageJ. *P < 0.05.

Figure S4

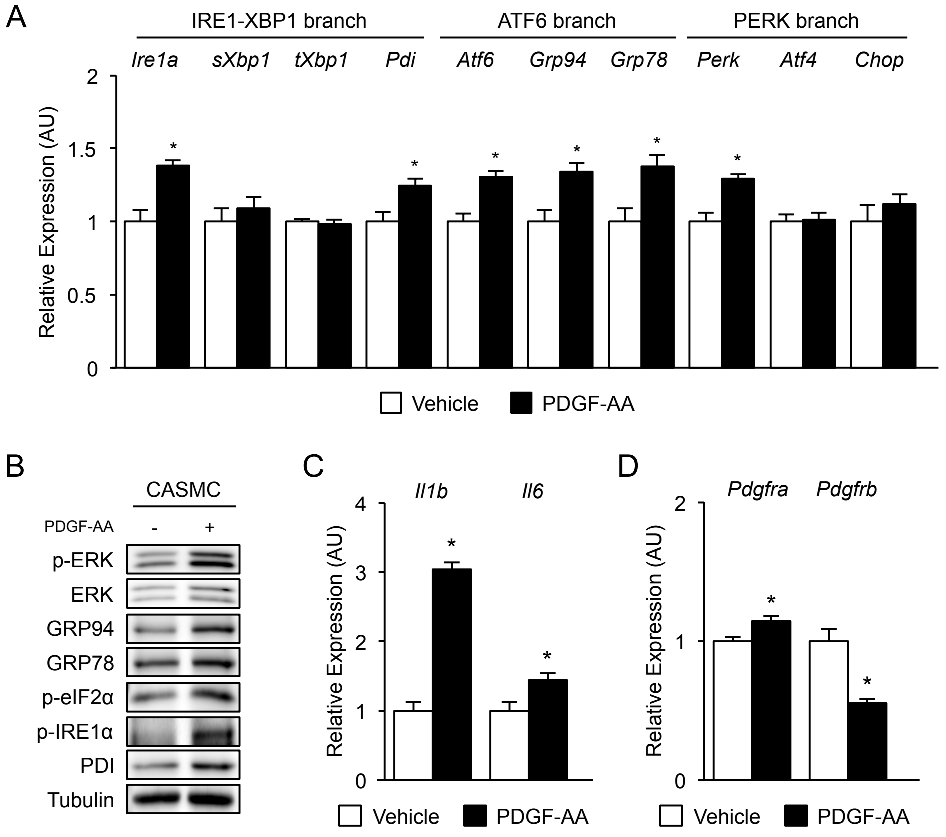

Figure S4. Effects of PDGF-AA on UPR activation and inflammation in CASMC.
A, B. Gene expression analysis (A) and Western blotting analysis (B) of ER stress markers in CASMC upon chronic stimulation with 20 ng/ml PDGF-AA for 48 h. **C, D.** Gene expression analysis of inflammatory cytokines (C) and PDGF receptors (D) in CASMC upon chronic stimulation with 20 ng/ml PDGF-AA for 48 h. *P < 0.05 vs. Vehicle.

Table S1

Table S1. Primers for quantitative real-time PCR for human genes

Genes	Species	Accession #		Forward primer			Reverse primer	
18s	Human	M10098	5'-	GTAACCCGTTGAACCCCATT	-3'	5'-	CCATCCAATCGGTAGTAGCG	-3'
Atf4	Human	NM_001675.2	5'-	ATGACCGAAATGAGCTTCCTG	-3'	5'-	CTGGAGAACCCATGAGGTTTG	-3'
Atf6	Human	NM_007348	5'-	TCAGACAGTACCAACGCTTATGC	-3'	5'-	GTTGTACCACAGTAGGCTGAGA	-3'
Chop	Human	NM_004083	5'-	GGAGAACCAGGAAACGGAAAC	-3'	5'-	TCTCCTTCATGCGCTGCTTT	-3'
Edem	Human	NM_014674	5'-	TCCAGAAAGCAGTCAAGTTAGTG	-3'	5'-	GAATCCCTGTCTTGGTGTTTTCA	-3'
Grp78	Human	NM_005347	5'-	CATCACGCCGTCCTATGTCG	-3'	5'-	CGTCAAAGACCGTGTTCTCG	-3'
Grp94	Human	NM_003299	5'-	CTGGGACTGGGAACTTATGAATG	-3'	5'-	TCCATATTCGTCAAACAGACCAC	-3'
Il6	Human	NM_000600	5'-	AAATTCGGTACATCCTCGACGG	-3'	5'-	GGAAGGTTCAGGTTGTTTTCTGC	-3'
Il1b	Human	NM_000576	5'-	CAC GAT GCA CCT GTA CGA TCA	-3'	5'-	GTT GCT CCA TAT CCT GTC CCT	-3'
Ire1a	Human	NM_001433	5'-	TTTGGAAGTACCAGCACAGTG	-3'	5'-	TGCCATCATTAGGATCTGGGA	-3'
Pdi	Human	NM_000918	5'-	GGTGCTGCGGAAAAGCAAC	-3'	5'-	TGATCTCGGAACCTTCTGCCT	-3'
Pdgfra	Human	NM_006206.4	5'-	AACCGTGTATAAGTCAGGGGA	-3'	5'-	GCATTGTGATGCCTTTGCCTT	-3'
Pdgfrb	Human	NM_002609.3	5'-	TCCAGCACCTTCGTTCTGAC	-3'	5'-	TATTCTCCCGTGTCTAGCCCA	-3'
Perk	Human	NM_004836	5'-	ACGATGAGACAGAGTTGCGAC	-3'	5'-	AATCCCACTGCTTTTTACCATGA	-3'
sXbp1	Human	AB076384	5'-	GGTCTGCTGAGTCCGCAGCAGG	-3'	5'-	GGGCTTGGTATATATGTGG	-3'
tXbp1	Human	NM_005080	5'-	AGTAGCAGCTCAGACTGCCA	-3'	5'-	CCTGGTTCTCAACTACAAGGC	-3'
